# Basics and applications of tumor-derived extracellular vesicles

**DOI:** 10.1186/s12929-019-0533-x

**Published:** 2019-05-11

**Authors:** Yu-Ling Tai, Pei-Yu Chu, Bao-Hong Lee, Ko-Chien Chen, Chia-Yu Yang, Wen-Hung Kuo, Tang-Long Shen

**Affiliations:** 1 Department of Plant Pathology and Microbiology, National Taiwan University, Taipei, Taiwan; 2 Department of Urology, University of Texas Southwestern Medical Center, Dallas, TX USA; 3 Division of Hematology and Oncology, Department of Internal Medicine, Taipei Medical University Hospital, Taipei, Taiwan; 4 Department of Surgery, National Taiwan University Hospital, Taipei, Taiwan; 5 Center for Biotechnology, National Taiwan University, Taipei, Taiwan

**Keywords:** Extracellular vesicles, exosome, tumor-derived EVs, homeostasis, tumor microenvironment, EV isolation, biomarker, drug delivery

## Abstract

Extracellular vesicle (EV)-mediated intercellular communication acts as a critical culprit in cancer development. The selective packaging of oncogenic molecules renders tumor-derived EVs capable of altering the tumor microenvironment and thereby modulating cancer developments that may contribute to drug resistance and cancer recurrence. Moreover, the molecular and functional characteristics of cancer through its development and posttreatment evolve over time. Tumor-derived EVs are profoundly involved in this process and can, therefore, provide valuable real-time information to reflect dynamic changes occurring within the body. Because they bear unique molecular profiles or signatures, tumor-derived EVs have been highlighted as valuable diagnostic and predictive biomarkers as well as novel therapeutic targets. In addition, the use of an advanced EV-based drug delivery system for cancer therapeutics has recently been emphasized in both basic and clinical studies. In this review, we highlight comprehensive aspects of tumor-derived EVs in oncogenic processes and their potential clinical applications.

## Background

### Basic characteristics of EVs

EVs are transportable vesicles that participate in the exchange of biological molecules between cells. They are pivotal in maintaining cellular and body homeostasis [1, 2]. The transfer of EVs serves as an efficient and specific delivery system that carries different types of cellular cargo, such as nucleic acids, lipids, proteins, and metabolites, to their target destinations [3]. Exosomes are defined as a specific subset of EVs that range from 30 to 150 nm in size. They are originally endosomal-derived intraluminal vesicles (ILVs) that are subsequently released into the extracellular milieu through the fusion of multivesicular endosomes or multivesicular bodies (MVBs) with the plasma membrane [4–6]. Exosomes retain a conserved series of proteins that are shared with the secreting cell during their biogenesis. However, their uniqueness stems from the variety of molecular constituents and lipid contents derived from their cell of origin and status. Given their intricate characteristics, exosomes display potent influence on recipient cells and show promises in revealing cell-to-cell communication.

In 1877, serum particles were investigated and described as “motes floating in the sunlight” by Edmunds [7]. Later, Peter Barland et al. investigated the structure of cellular vesicles under an electron microscope [8]. However, the function of these cellular vesicles remained unclear until 1967, when Peter Wolf identified lipid-rich particles that displayed coagulant properties that he suggested had originated from the granules of platelets [9]. In 1981, the term “exosome” was first coined to describe extracellular vesicles with an average diameter of 500 to 1000 nm [10]. Later, the Johnstone group and the Stahl team independently reported that bioactive molecules shed from reticulocytes, such as transferrin receptors, were incorporated within vesicles (approximately 50 nm in diameter) and released by exocytosis in MVBs [11–13]. In 1987, Rose M. Johnstone et al. further described the functional link between exosomes and reticulocyte maturation [6]. Although exosomes are secreted by a wide range of mammalian cell types [4, 5], exosomes enclose limited cytosol from their parent cells with their lipid bilayers and are devoid of cellular organelles. The compositions of exosomes reflect the physiological and/or pathological states of their parent cells and are associated with their environmental conditions and/or stimuli [14]. Moreover, the distinct contents of exosomes heavily depend on their parent cell types and functions, which suggests that exosomes have the unique property of cargo selectivity [15]. Currently, exosomes can be isolated from almost all types of cells and various physiological and pathological fluids, such as blood, saliva, milk, urine, cerebrospinal fluid, ascites, tears, and pleural effusions [16–18].

Indeed, exosomes, the predominant form of microvesicles, are stable and abundant in bodily fluids (> 10^9^ vesicles/mL of blood) [19]. Cancer cells especially secrete more exosomes than healthy cells [20], suggesting that exosomes function as critical mediators of cancer development.

### EVs in homeostasis

EVs maintain cellular homeostasis by transporting bioactive and/or regulatory molecules between cells and tissues. For instance, exosome secretion ablates the harmful cytoplasmic accumulation of nuclear deoxyribonucleic acid (DNA) in cells by preventing the aberrant innate immune response [21]. Consistently, the inhibition of exosome secretion, such as the depletion of alpha-1,3/1,6-mannosyltransferase (ALG2)-interacting protein X (Alix) or Rab27a, induced the cytoplasmic accumulation of nuclear DNA and subsequently activated a stimulator of the interferon genes, a cytoplasmic double stranded DNA (dsDNA) sensor, contributing to the reactive oxygen species-dependent DNA damage response [21]. In neural EVs, cysteine string protein α, which regulates refolding pathways at the synapse, is involved in the EV-mediated cellular export of disease-associated proteins such as polyglutamine expanded protein 72Q huntingtin^ex°n1^ or superoxide dismutase-1^G93A^ [22]. In contrast, a loss-of-function mutation of cysteine string protein α ablated the EV-mediated cellular export of disease-associated proteins [22], suggesting the critical role of the EV-mediated removal of toxic proteins in neurons.

The interaction between receptor activator of nuclear factor-κB-ligand (RANKL)/ receptor activator of nuclear factor -κB (RANK) induces osteoclast differentiation and function in bone homeostasis. Osteoblast-derived EVs have been shown to participate in this process by transferring RANKL to osteoclast precursors to promote osteoclast formation [23]. In contrast, RANK-enriched EVs regulated bone homeostasis by competing with RANK to interact with RANKL on the surfaces of osteoclasts [24]. Recently, maturing osteoclasts-derived small EVs that contain RANK have been shown to induce RANKL reverse signaling in osteoblasts via the activation of Runt-related transcription factor 2 to facilitate bone formation [25]. Together, these studies indicate the importance of EVs in the maintenance of the homeostatic cellular balance.

### EVs in cancer communication

EVs regulate the dynamic and functional communication between cancer stem cells and cancer cells/the tumor microenvironment during cancer development [26]. Indeed, exosomes secreted by C-X-C chemokine receptor type 4 (CXCR4)-overexpressing breast cancer cells exhibited high levels of stemness-related markers and metastatic-related messenger ribonucleic acids (mRNAs) [27]. Moreover, recipient cells treated with exosomes derived from CXCR4-overexpressing cells also demonstrated high expression levels of stemness-related markers and an increase in the invasive ability and metastatic potential of cancer cells [27]. Moreover, adipose-derived mesenchymal stem cells secrete exosomes to facilitate cancer migration and proliferation in a wingless/integrated (Wnt)/β-catenin signal-dependent manner [28]. In colorectal cancer, exosome derived from cancer-associated fibroblasts have been shown to prime cancer stem cells and to contribute to drug resistance and chemoresistance through Wnt signaling [29]. Additionally, the chemotherapeutic agent gemcitabine induced the upregulation and secretion of miR-146a and Snail in cancer-associated fibroblast-derived exosomes, facilitating proliferation and drug resistance in recipient pancreatic cancer cells [30]. During gemcitabine treatment, the inhibition of exosome generation by the inactivation of neutral sphingomyelinase significantly reduced the survival of cocultured pancreatic cancer cells [30]. Fibroblast-derived exosomes that contain Wnt have been shown to contribute to chemotherapy resistance by restoring cancer stem cell characteristics in colorectal cancer cells in a Wnt/β-catenin signal-dependent manner [31], suggesting an important effect of EVs derived from the tumor microenvironment on drug resistance in cancer. Moreover, chemotherapeutic drugs, such as paclitaxel or doxorubicin, elicited EVs enriched in annexin A6 that were shown to active endothelial cells, induce pulmonary C-C motif chemokine ligand 2 expression, and increase C-C chemokine receptor type 2-positive monocyte expansion, leading to mammary tumor metastasis [32]. These studies indicated the critical role of EVs in cell-to-cell communication during cancer development.

### Association between tumor-derived EVs and cancer development

Tumor-derived EVs with protumorigenic activity regulate cancer development by promoting cancer aggressiveness, cancer invasiveness, the remodeling of the extracellular matrix, angiogenesis, drug resistance, and immunosuppression [14, 33], suggesting the important effects of tumor-derived EVs on cancer development and cancer therapy.

The transfer of metastatic components (i.e., oncogenic proteins or oncogenic microRNAs, oncomiRs) can trigger and reprogram signaling cascades, phenotypes, and the functions of recipient cells [34, 35]. Tumor-derived EVs, especially exosomes, with prometastatic effects can also govern the pathogenesis of cancer invasion and metastasis. For example, exosomal cluster of differentiation 44 (CD44) is transferred from ovarian cancer cells to peritoneal mesothelial cells, which subsequently results in the mesenchymal and spindle morphology of peritoneal mesothelial cells and contributes to cancer invasion [36]. Indeed, numerous studies have shown exosomes to be involved in epithelial–mesenchymal-transition (EMT) during the development of malignant cancer [37]. The uptake of pancreatic cancer-derived exosomes by Kupffer cells elicited premetastatic niche formation through increasing transforming growth factor β (TGF-β) secretion and fibronectin expression by hepatic stellate cells and subsequently promoted liver metastasis [38, 39]. Moreover, tumor-derived exosomal miR-1247-3p has been shown to activate the β1-integrin/nuclear factor kappa-light-chain-enhancer of activated B cells (NF-κB) signaling axis, resulting in the activation of cancer-associated fibroblasts to foster the metastasis of liver cancer to the lung [34]. The transfer of EV microRNAs, such as miR-200, from highly metastatic breast cancer cells to poorly metastatic cells altered gene expression and facilitated mesenchymal-to-epithelial transition, which promoted metastasis within the weaker metastatic cells [40]. Moreover, highly metastatic melanoma-derived exosomes exhibited a prometastatic phenotype caused by an increase in MET expression in educating bone marrow progenitor cells [41]. Indeed, exosomes derived from highly metastatic melanomas altered their metastatic activity to produce poorly metastatic melanomas [42], implicating the strong connection between exosomes and cancer malignancy. Furthermore, tumor-derived exosomes promoted hypoxia-driven pro-angiogenic tumor responses [43] during cancer development, indicating the importance of tumor-derived exosome in the regulation of sustained angiogenesis. The transfer of miR-130a from gastric cancer cells to vascular endothelial cells through exosomes facilitated angiogenesis and cancer growth by targeting *c-MYB* [44]. It is believed that EVs function as critical mediators of cancer development and malignancy. The following describes in detail the contents of EVs, their isolation and the analytical approaches used in tumor-derived EV studies.

### EV contents

**Figure 1:**
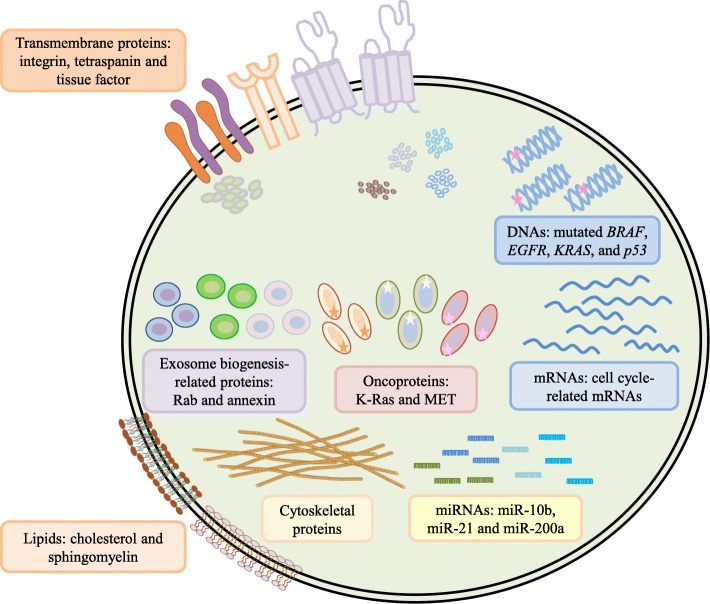
Summary of diverse bioactive molecules in tumor-derived EVs. Tumor-derived EVs are phospholipid bilayer-enclosed vesicles that contain diverse bioactive molecules. These bioactive molecules can be divided into general groups, such as nucleic acids, proteins, lipids, and metabolites. The nucleic acid group contains mutated oncogenes/ tumor suppressor genes, cell cycle-related mRNAs, and cancer-related miRNAs. The protein group can be divided into several subgroups: transmembrane proteins, growth factors, exosome biogenesis-related proteins, oncoproteins, and cytoskeletal proteins. Among members of the lipid group, cholesterol, sphingomyelin, prostaglandins, and leukotrienes can be detected in EVs. Some amino acids, pyruvate, lactate, and TCA-cycle intermediates are included in the metabolite group

EVs containing varied molecular cargos (Figure 1), such as nucleic acids (i.e., DNAs, mRNAs, microRNAs, long noncoding RNAs and many noncoding RNAs), proteins, lipids, and metabolites, are directly internalized by recipient cells, leading to morphological and functional changes in the recipient cells [14, 33]. It has been highlighted in recent years that the transfer of oncogenic cargo through EVs and tumor-derived exosomes drives oncogenic signal transduction cascades in association with the development of cancer malignancies and tumor microenvironments [5, 35]. Table 1 lists the functional effects of EV cargos during cancer development.

**Table 1:** Functional effects of EV cargos in cancers

EV cargo	Type of cargo	Functional effects	Reference
Amino acids or TCA-cycle intermediates	Metabolites	Promote cancer growth	[74]
Annexin A6	Protein	Pre-mPremetastatic niche formation	[31]
Integrins	Protein	Organotropic metastasis	[39]
Lethal-7 miRNA family	miRNA	Maintain the highly metastatic tumorigenic phenotype	[56]
MET	Protein	Pre-mPremetastatic niche formation	[41]
miR-223	miRNA	Enhance cancer invasion	[57]
miR-10b and miR-21	miRNA	Regulate cancer development	[59]
Mitochondrial DNA		Regulate escape from dormancy in therapy-resistant cancers	[47]
Mutant K-Ras	Protein	Enhance three-dimensional growth of cells	[68]
TGF-β	Protein	Promote fibroblast-myofibroblast differentiation	[64, 65]
Tissue factor	Protein	Regulate pro-coagulant activity of endothelial cells	[66]

#### DNAs

Oncogenes and tumor suppressor genes are key mediators during cancer progression and malignancy. Several studies have indicated that dsDNAs represent the largest proportion of exosomal DNAs (exoDNAs) in tumor-derived exosomes [45]. Indeed, double-stranded genomic DNA spanning all chromosomes has been detected in exosomes [46]. Moreover, fragments of mutated *KRAS* and *p53*, the most frequently mutated oncogenes or tumor suppressor genes, within exosomes derived from pancreatic cancer cells have been investigated in clinical studies [46]. Additionally, exoDNAs contain similar mutations, such as *BRAF* (*V600E*) and mutated epidermal growth factor receptor (*EGFR*), as the cancer cell lines from which they originated [45], suggesting the potential role of exoDNAs as alternative biomarkers in the detection and diagnosis of cancers. Additionally, the complete circular mitochondrial genome packaged within exosomes has been shown to regulate exit from therapy-induced metabolic dormancy in hormonal therapy-resistant breast cancer [47]. Nevertheless, how genomic DNA or mitochondrial DNA is packaged into exosomes and the regulatory mechanisms or functional consequences of exosomal DNA in recipient cells remain controversial. Indeed, studies reported that retrotransposon elements present in EVs potentially had a genetic influence on disease development with low efficiency [48, 49].

#### mRNAs

mRNAs within tumor-derived EVs influence the translational profiles of recipient cells during tumor progression [50, 51]. In 2008, Skog et al. showed that functional mRNAs incorporated into EVs were delivered to and translated by recipient cells [50]. Functionally, EVs containing mRNAs for oncogenic proteins were enriched in angiogenic proteins and induced tubule formation in recipient endothelial cells [50]. Additionally, the enrichment of cell cycle-related mRNAs leading to endothelial cell proliferation was investigated in the transcriptome of human colorectal cancer-derived EVs [52]. Of note, fewer than one copy of nonribosomal RNA was indicated per EV, suggesting that the uptake of abundant tumor-derived EVs as well as EV-RNAs by recipient cells might be essential for the functional effects of EV-RNAs on recipient cells [53]. Mutant mRNA variants and miRNAs signatures found in glioblastoma-derived EVs were clinically detected in only patients with glioblastoma [50]. These studies emphasize that tumor-derived EVs with the oncogenic characteristics of the host serve as effective biomarkers for cancer diagnosis and potential therapeutic targets.

#### MicroRNAs

MicroRNAs (miRNAs) are small noncoding RNAs that inhibit gene expression by binding to the 3’ untranslated regions (UTRs) of messenger RNAs, which subsequently leads to mRNA destabilization, translational inhibition, or mRNA degradation [54]. During physiological and pathological processes, the transfer of miRNAs (i.e., oncomiRs) through EVs mediates cell-to-cell communication. Indeed, the encapsulation of miRNAs within exosomes protects miRNAs from degradation, thereby increasing the diagnostic value of miRNAs contained in exosomes in cancer pathogenesis [55].

Several studies have investigated the enrichment of selective miRNAs in EVs in malignant cancers. For example, Ohshima et al. found that members of the enriched lethal-7 (let-7) miRNA family, tumor suppressors targeting oncogenic Ras, were selectively enriched in only highly metastatic gastric cancer-derived exosomes, leading to the maintenance of a highly metastatic tumorigenic phenotype by exosome-mediated clearance [56]. Additionally, tumor-associated macrophages (TAMs) release EVs containing miRNAs (i.e., miR-223) that enhance the invasiveness of breast cancer through the myocyte enhancer factor 2C/β-catenin pathway [57]. Since angiogenesis facilitates the process of cancer malignancy, angiogenesis regulated by EV miRNAs has been highlighted in recent studies [58]**.** EVs released from renal cancer stem cells stimulated angiogenesis through the upregulation of angiogenic factors (i.e., vascular endothelial growth factor) or extracellular matrix (ECM) degradation/remodeling enzymes (i.e., matrix metallopeptidase 2 (MMP2) and MMP9) in premetastatic lung niches, leading to lung metastasis [58]. Of note, the association between miRNAs (i.e., miR-10b and miR-21) and the RNA-induced silencing complex-loading complex in breast cancer-derived exosomes processes precursor miRNAs into mature miRNAs in a cell-independent manner, resulting in cancer development [59].

Interestingly, the opposite effect of miRNAs within EVs during cancer development was observed in glioblastoma. The pro-oncogenic effects of tumor-derived EVs were ablated by miR-1 which targets the abundant protein annexin A2 in glioblastoma-derived EVs, leading to tumor suppression of the glioblastoma microenvironment [60]. This finding provides an alternative strategy for miRNA-based targeted therapy for cancer treatment.

#### Proteins

The notion that several enlisted proteins are selectively packaged into EVs rather than packaged through a random process is commonly accepted today. Unlike membrane vesicles released by apoptotic cells with limited amounts of bioactive proteins, exosomes are enriched with varied bioactive proteins originating from the plasma membrane (i.e., growth factor receptors, integrins and tetraspanins), the cytosol (e.g., Rabs and annexins) and other intracellular compartments dependent on the endocytic pathway [61, 62].

Integrins are a major family of cell surface receptors that mediate cell adhesion to the ECM and modulate the bidirectional integration of signals between the inside and outside of a cell. Similarly, exosomal integrins exhibit an adhesive function by directing exosomes to recipient cells [62, 63], rendering specific and efficient intercellular communication. Of note, tumor-derived exosomal integrins have been reported to be highly associated with the metastasis of human breast cancer to the lung [39], resulting in organotropic metastasis. Targeting integrin within tumor-derived exosomes can interrupt exosome uptake by recipient cells and ablate cancer metastasis [39], implicating the potency of exosomal integrins in targeted cancer therapies.

Various studies have indicated that tumor-derived EV proteins shape the tumor microenvironment by remodeling the ECM, re-educating stromal cells, or activating angiogenesis, thereby facilitating cancer development. The transfer of growth factors (i.e., TGF-β) by tumor-derived exosomes influences the procancer stromal environment by elevating α-smooth muscle actin expression and promoting fibroblast-myofibroblast differentiation [64]. TGF-β-containing exosomes triggered fibroblast differentiation that supports angiogenesis and accelerates tumor progression [65]. Furthermore, mesenchymal-like cancer-derived EVs that exhibited an upregulated tissue factor, a transmembrane receptor for the coagulation factor VII/VIIa, affected the procoagulant activity of endothelial cells and led to cancer malignancy [66].

Importantly, tumor-derived exosomes carrying oncoproteins (i.e., mutant K-Ras) have been investigated in several types of cancers, such as human colorectal cancer [67, 68]. The transfer of mutant K-Ras from mutant K-Ras-expressing cells into nontransformed recipient cells by tumor-derived exosomes enhanced the three-dimensional growth of the nontransformed recipient cells [68]. Additionally, metastatic melanoma-derived exosomes transferred the MET oncoprotein to bone marrow-derived cells, enabling the promotion of premetastatic niche formation and indicating the importance of tumor-derived exosomes with a cancer metastasis-related protein signature [41].

#### Lipids and metabolites

Different types of lipids, such as cholesterol, diglycerides, sphingolipids, phospholipids, polyglycerophospholipids, and phosphatidylethanolamine, are predominantly expressed in EVs [69, 70]. Cholesterol, sphingomyelin, phosphatidylserine, and phosphatidylinositol especially promote EV membrane rigidity [69]. Some bioactive lipids (i.e., prostaglandins and leukotrienes) and lipid metabolism-related enzymes have also been detected in EVs [69, 71, 72], suggesting the potential role of EVs with cancer progression-related lipids in cancer development.

The intrinsic metabolic activity of EVs has demonstrated their ability to synthesize adenosine triphosphate by glycolysis as well as carry varied metabolites and metabolic enzymes, including pyruvate, lactate, and lactate dehydrogenase isoforms [73]. Thus, the metabolism of the recipient cells is altered by the uptake of EVs. Of note, a study by Zhao et al. [74] demonstrated that exosomes derived from cancer-associated fibroblasts provided diverse metabolites, such as amino acids or tricarboxylic acid (TCA)-cycle intermediates, to nutrient-deprived cancer cells to promote prostate or pancreatic cancer growth in a K-Ras independent manner.

### Isolation of EVs

To understand how EVs participate in physiological and pathological processes, an efficient and reliable strategy for the isolation of EV with high purity — low contamination from other extracellular vesicles, soluble proteins, or broken cells — is challenging but necessary for basic experimental and clinical analyses. Based on the physical properties of exosomes, which have a specific buoyant density and different in flotation velocities, differential centrifugation is the most common method to isolate exosomes from cell culture conditioned media or physiological fluids [4, 75–77]. Accordingly, live or dead cells, cellular debris, and large particles in the cell culture conditioned media or physiological fluids are first separated by gradual centrifugal forces between 200 × g to 10,000 × g, followed by the application of ultracentrifugal force at 100,000 × g to isolate exosomes [75]. In the basic research setting, the most widely used sample for EV isolation is conditioned cell culture media [77]. Larger sample volumes are required for the isolation of EVs from cell culture conditioned media than the isolation of EVs from biofluids, such as plasma, serum, or urine [77]. Although some concerns, such as the time-consuming nature and high equipment cost for EV isolation, have been addressed, the ultracentrifugation method allows for EV isolation from large sample volumes and produces high yields of EVs [77, 78]. However, the differential centrifugation method often results in protein aggregates or contaminations with particulates with similar physical properties in the isolated EVs [75]. To resolve these impurities, modified EV isolation methods combining differential centrifugation and immunoadsorption techniques [79] or sucrose gradient ultracentrifugation [80] have been reported in several studies.

Due to increasingly high demand from the emerging field of EV-based therapeutics and diagnostics, several methods and commercially available kits for EV isolation based on size exclusive chromatography, microfluidics, immunoaffinity, or flow metrics provide easy and efficient methods for the enrichment of purified EVs from liquid samples, such as cell culture conditioned media or physiological fluids. In fact, the principle of these methods is separation according to the density, size, mass, surface charge and/or surface protein features of EVs. Sized-based isolation methods mainly depend on the size or molecular weight of EVs. For example, ultrafiltration combined with sequential filtration, which is a faster procedure than ultracentrifugation despite the observation of large vesicle deformation, is used for exosome isolation [81]. Ultrafiltration, which has no special equipment requirements, is more efficient than ultracentrifugation [82]. In clinical studies, a nanomembrane ultrafiltration concentrator was used to rapidly isolate exosomes from human urine samples [82]. Alternatively, size exclusive chromatography, which is easy and fast, to isolate EVs or exosomes from conditioned media or plasma samples depends on the hydrodynamic radius of the exosomes; nanoscale exosomes enter most of the porous beads and can be collected in the latter fractions, unlike larger particles, such as microvesicles or apoptotic bodies [83, 84].

Regarding precision in exosome isolation, immunoaffinity-based methods exhibit high specificity for exosome isolation. Accordingly, these methods rely on specific exosomal surface proteins, such as CD9, CD63, or CD81, that can be captured by their corresponding antibodies [85]. Although immunoaffinity-based methods are not suitable for EV isolation from large sample volume, high-purity EVs can be isolated from the conditioned media of cancer cells or plasma samples from patients with cancer [85–87]. Notably, exosomes purified by immunoaffinity-based methods only represent a subpopulation of the whole exosome population and exhibit various characteristics of the subpopulation corresponding to different antibodies, even when the antibody exhibits the same specificity against the same surface protein. Exosome precipitation involves altering the solubility or dispersibility of exosomes. The easy, rapid, and cost-effective method of EV isolation by polyethylene glycol is often employed to separate exosomes from the original soluble samples to discharge water molecules and force exosomes out of solution [81, 88]. In a longitudinal study, polyethylene glycol-based precipitation enriched EVs from human serum samples for subsequent EV miRNA analysis [89]. Inevitably, EVs purified with this method coprecipitate with contaminants, such as membrane-free macromolecular/protein aggregates or particulates, which results in impure isolated exosome populations.

In the clinical setting, it is very important to isolate EVs from small sample volumes within a fairly limited time period to improve the clinical potential of EVs in cancer diagnosis [90]. Numerous innovative methods of EV isolation, such as microfluidic- or flow cytometry-based methods, have been developed to enhance the sensitivity and specificity of EV isolation to fulfill clinical requirements [90]. Microfluidic-based EV purification methods, including sieving EVs from blood samples through nanoporous membranes [91], capturing EVs from clinical plasma samples by an immunoaffinity antibody [92–94], or trapping EVs on porous structures such as porous silicon nanowire-on-micropillar structures, are efficient and fast [95]. Notably, microfluidic-based methods require additional off-chip processes for sample precleaning/preparation and/or reagent mixing [93]. Due to the size limitation of detection by forward scattered light, magnetic beads coated with antibodies or commercial fluorescent-labeled antibodies against exosomal surface biomarkers allow exosomes to be detected and isolated by flow cytometry [96, 97]. Despite its low yields and high costs, a newly developed method based on the characterization, size-based separation, and quantification of exosomes by the asymmetrical flow field-flow fractionation (AF4) technique combined with multidetection systems, such as ultraviolet (UV) or multiangle light scattering (MALS) [98, 99], displays the powerful ability to produce high-purity EV subpopulations, such as large exosome vesicles, small exosome vesicles and exomeres, at a high resolution [100]. Table 2 summaries the pros and cons, such as yield, purity, processing time, cost, and standardization, of the current methods commonly utilized for EV isolation.

**Table 2:** Summary of common EV isolation methods

Method	Description	Example	Yield	Purity/quality	Equipment/Cost	Approximate processing time	Commercial products	Reference
Ultracentrifugation-based methods	Size- or density-dependent isolation	1. Differential centrifugation/Ultracentrifugation 2. Sucrose gradient ultracentrifugation	High	Protein aggregates and particulates contaminations	High equipment cost	2.5 – 48 h	No commercial available	[75, 77–80]
Size-based methods	Size- or molecular weight-dependent isolation	1. Ultrafiltration 2. Size exclusive chromatography (SEC) 3. Asymmetrical-flow field-flow fractionation (AF4)	Low (AF4)	High purity (AF4, SEC); exosomes may be deformed (ultrafiltration)	Low/moderate equipment cost (ultrafiltration and SEC ); high equipment cost (AF4)	1 - 1.5 h (Ultrafiltration); 0.5 - 1 h (SEC); 1h (AF4)	Available, .e.g.. EVSecond column (GL Sciences) or qEV column (Izon Science)	[82–84, 98–100]
Immunoaffinity-based methods	Antibody-antigen interaction-dependent isolation	1. Immunocapture 2. Immunoadsorption	Low	High purity	High reagent cost	4 - 5 h	Available, e.g. MagaCapture™ Exosome Isolation Kit (Wako) or Exosome-Human CD9 Isolation Reagent (ThermoFisher)	[85–87]
Precipitation-based methods	Solubility- or dispersibility-dependent isolation	Polyethylene glycol	Dependence	Contaminated precipitates	Low equipment cost	1 h	Available, e.g. Total Exosome Isolation Kit (Invitrogen) or ExoQuick™ Exosome Precipitation (System Biosciences)	[81, 88, 89]
Microfluidic-based methods	Size-, density-, or antibody-antigen interaction-dependent isolation	Microfluidic device with nanoporous membrane, immuno-chip, or porous silicon nanowires-on-micropillar structure	Dependence	High purity (porous nanowires-on-micropillar structure); EVs may be damaged (nanoporous membrane-based filtration)	Low/moderate equipment cost	2 h (porous membrane-based filtration); 1.5 h (immuno-chip)	No commercial available	[91–95]
Flow cytometry-based methods	Antibody-antigen interaction-dependent isolation	1. Fluorescent-labeled antibody-based isolation 2. Immuno-magnetic-based isolation	Low	High purity	High equipment and reagent cost	12 h	Avaialbe, e.g. Exosome Flow Cytometry Kit (Wako)	[96, 97]

### Characterization of EVs

In basic experimental and clinical studies, the characterization of isolated EVs by imaging, biochemical techniques, or physiochemical techniques is an essential step. The main methods used to characterize isolated EVs are electron microscopy, nanoparticle tracking analysis, Western blotting, and flow cytometry. Due to their nanometer size, the morphology of exosomes is subject to visualization and further determination by electron microscopy (EM). Typically, the rounded structure of exosomes is investigated by cryogenic electron microscopy [101]. Moreover, a modified method to characterize exosome, the immuno-EM method, which combines electron microscopy and antigen-specific immunolabeling, has been reported in many basic experimental studies [75]. Furthermore, nanoparticle tracking analysis (NTA, a NanoSight^TM^ technology) based on the Brownian motion of particles allows for the determination of the size distribution and particle concentration of EVs [102]. Other commercial NTA systems with similar outputs are available on the market. According to the molecular composition of EVs, separation of EV proteins by SDS-PAGE, followed by immunoblotting for specific EV biomarkers, such as CD9, CD63, CD81, heat shock protein 70 (HSP70), HSP90, or Alix, enables the characterization of EVs [103]. Additionally, flow cytometry-based methods, such as AF4/UV-MALS, are used to determine the size and amount of the particles discussed earlier [100].

Notably, the International Society for Extracellular Vesicles (ISEV) has recently launched the “Minimal Information for Studies of Extracellular Vesicles 2018 (MISEV2018)” guideline [104] by updating the MISEV2014 guideline to provide comprehensive mandatory considerations for EV characterization, including quantification, global characterization, and single EV characterization.

### Roles and potential applications of tumor-derived EVs

**Figure 2:**
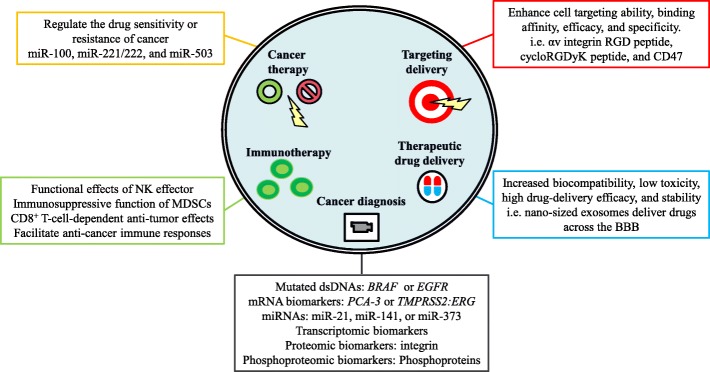
Potential applications of tumor-derived EVs. Investigations of the specific expression patterns of mRNAs/miRNAs and transcriptomic, proteomic, and phosphoproteomic biomarkers in tumor-derived EVs, have indicated a powerful role for tumor-derived EVs in cancer diagnosis. The functional effects of tumor-derived EVs on the regulation of drug sensitivity or resistance in cancer suggest the role of tumor-derived EVs in cancer therapy. Regarding the role of EVs in immunotherapy, exosomes have been shown to modulate NK effector functional effects, reinforce the immunosuppressive function of MDSCs, and facilitate anticancer immune responses. A growing number of studies have indicated that the RGD peptide, cyclic RGDyK peptide, and CD47-modified exosomes promote the cell targeting, binding affinity, efficacy, and specificity of exosomes, suggesting the potential applications of EVs in targeted delivery. Moreover, the EV-based therapeutic drug delivery system exhibits increased biocompatibility, low toxicity, and stability

The pathologic functions of tumor-derived EVs in cancer development and malignancy are highlighted by their *bona fide* effects; EVs facilitate transformation, survival, immunosuppression, epithelial-mesenchymal transition (EMT), invasion, angiogenesis, premetastatic niche formation, and metastasis. Understanding tumor-derived EVs renders a new avenue to monitor and treat cancer since cancer evolves over time during cancer development or treatment. The dynamic and comprehensive molecular information within tumor-derived EVs reflects *de novo* cancer evolution, which also sheds light on novel and valuable diagnostic and prognostic factors. Overall, the potential and potent applications of tumor-derived EVs will fulfill the fundamental needs of and strategies for precision medicine. The illustration of Figure 2 encompasses the potential roles and applications of tumor-derived EVs.

### EVs in cancer diagnosis

The fundamental basis of precision medicine was recently highlighted by biomarker discovery through liquid biopsy, which allows for noninvasive, fast, dynamic, low-cost, and accurate diagnosis for the early and real-time detection of cancer. To gain comprehensive information on cancer development and progression, the use of several potential and meaningful biomarkers, such as circulating tumor cells (CTCs), cell free DNAs (cfDNAs), and EVs, in liquid biopsy aimed at the clinical detection of various types and stages of cancers has been intensively investigated over the past two decades [105]. Taking advantage of their highly dynamic and multispecies nature, abundance, and stability, cancer-related EVs have served as suitable and precise biomarkers in various clinical settings. Indeed, the exosomal protein tumor susceptibility gene 101 (TSG101) stored at -20 °C or -80 °C was stable for over 3 months [106]. Moreover, phosphoproteins from microvesicular and exosomal EVs from human plasma were stable for up to 5 years [107]. Notably, although CTCs and cfDNAs allow for the rapid and easily accessible diagnosis of malignant cancers, the limitations in detecting late-stage malignancies and the technical challenges of cfDNAs and CTCs have been indicated in several clinical utilities. Although cfDNAs enable the detection of tumor-specific mutations [108], the expeditious elimination of DNA by nucleases has been documented [109]. Although double-stranded DNA remains in the blood longer than single-stranded DNA, the rapid clearance of DNAs is indispensable regardless of its size or strandedness, which limits the application of cfDNA in cancer diagnosis [110]. Due to the rarity of CTCs in the bloodstream and the lack of meaningful information on the number of CTCs in patients’ circulating blood [111], improvements in detecting and capturing CTCs is required for the use of CTCs for clinical diagnosis.

#### EV miRNAs in cancer diagnosis

Versatile bioactive molecules enclosed within EVs are protected from degradation [112]. With this in mind, the content of EVs was profiled to decipher biomolecules with mechanistic and/or diagnostic specificity for varied types of cancers; because of their consistent and robust detection, these biomolecules are regarded as valuable biomarkers in liquid biopsy for cancer diagnosis and prognosis (Table 3). Consistently, in an RNA-based clinical analysis, exosomes protected miRNAs from degradation by ribonucleases in feces [121]. Indeed, miRNA expression profiling in circulating EVs, such as exosomes, has been performed to establish the predictive function of specific miRNA signatures in human peripheral blood [122]. Recent efforts have also been devoted to the discovery of early diagnostic biomarkers for cancer due to the significant improvement in the survival of cancer patients. For example, miR-21, miR-141, miR-200a, miR-200b, miR-200c, miR-203, miR-205, and miR-214 were significantly elevated in exosomes from patients with ovarian cancer compared to those from patients with benign disease [116]. Additionally, exosomes derived from the serum of patients with hormone receptor-negative breast cancer (estrogen receptor-negative or progesterone receptor-negative) exhibited a higher level of miR-373 expression than those with the serum of patients with hormone receptor-positive breast cancer [115]. Moreover, upregulation of the exosome miR-21 in serum from patients with esophageal squamous cell carcinoma was highly correlated with advanced tumor classification, positive lymph node status, and metastasis [114], suggesting that EV miRNAs provide useful diagnostic information to evaluate the status of cancer development. Importantly, the development of EV-based liquid biopsy from saliva and urine provides an alternative, noninvasive and sensitive strategy for cancer detection. In this technique, exosomes derived from the saliva of mice with pancreatic cancer exhibited pancreatic cancer-specific salivary transcriptomic biomarkers [118]. The inhibition of exosome biogenesis altered this pancreatic cancer-specific transcriptomic biomarker profile in salivary exosomes [118]. Clinically, exosomes derived from the urine of patients with prostate cancer have also been documented to display specific prostate cancer mRNA biomarkers, such as prostate cancer antigen 3 and transmembrane protease serine 2:transforming protein ERG (*TMPRSS2:ERG*) [117].

**Table 3:** EV cargos used as diagnostic biomarkers in cancers

EV cargo	Type of cargo	Type of body fluid	Cancer type	Reference
CD63 and caveolin-1	Protein	Plasma	Melanoma	[113]
Integrin	Protein	Plasma	Breast cancer	[39]
miR-21	miRNA	Serum	Esophageal squamous cell carcinoma	[114]
miR-373	miRNA	Serum	Breast cancer	[115]
miRNA signatures	miRNA	Serum	Ovarian cancer	[116]
Prostate cancer antigen 3 and *TMPRSS2:ERG*	RNA	Urine	Prostate cancer	[117]
Salivary transcriptomic biomarkers	RNA	Saliva	Pancreatic cancer	[118]
Specific phosphoproteins	Protein	Plasma	Breast cancer	[107]
Specific protein profile	Protein	Ascites	Colorectal cancer	[119]
Survivin	Protein	Plasma	Prostate cancer	[120]

#### EV proteins in cancer diagnosis

Cancer-specific EVs carry specific and stabile protein cargo for intercellular signal exchange to regulate the tumor microenvironment. Likewise, the detection of unique EV proteins associated with cancer development and progression has been emphasized in various clinical utilities (Table 3). For instance, survivin expression was significantly higher in plasma exosomes from patients with prostate cancer compared to that in plasma exosomes from patients with pre-inflammatory benign prostatic hyperplasia or normal healthy controls [120]. Utilizing proteomic analysis, the specific protein expression profiles in diverse body fluids EVs, including ascites from colorectal cancer patients, were illustrated and determined [119]. Increased CD63 or caveolin-1 was detected in plasma exosomes from patients with melanoma compared to that in plasma exosomes from healthy donors [113]. Recently, our study indicated that tumor-derived exosomes with specific integrin expression profiles regulated organotropic metastasis [39]. This was the first study to show that bioactive molecules in exosomes could determine and predict the specific organ of cancer metastasis, further implicating exosome integrin profiles as biomarkers for organotropic metastasis [39]. Given that protein phosphorylation is essential in many cancer cell functions, the phosphoproteome analyses of tumor-derived EVs from human plasma provide valuable information for cancer diagnosis. Specific phosphoproteins in plasma EVs are significantly increased in patients with breast cancer compared to those in healthy controls [107]. Together, these studies suggest that the profiles of specific bioactive molecules in tumor-derived EVs function as novel and valuable biomarkers to diagnose or track the real-time status of cancer during cancer development and progression.

### EV biology during cancer therapy

Given that neoadjuvant chemotherapy and chemoradiotherapy are effective anticancer therapeutic strategies in many types of cancers, understanding the detailed mechanisms of the pathologic changes in response to therapy is essential to optimize preoperative and postoperative treatments. Because they manage both bioactive molecules and cellular waste in cells, therapeutic treatment-induced EVs reflected the response of cancer cells upon encountering anticancer treatments [123]. Indeed, cisplatin-resistant cell-derived exosomes contained more platinum than those derived from cisplatin-sensitive cells [124]. Moreover, exosomes have been shown to regulate the cisplatin sensitivity of lung cancer [125]. Clinically, the secretion of annexin A3 is associated with exosomes released from patients with platinum-resistant ovarian cancer [123]. Together, these studies suggest the functional effects of EVs on the regulation of drug sensitivity and response.

The EV-mediated transfer of miRNAs has also been attributed to drug resistance. For instance, drug-resistant breast cancer-derived exosomes regulated the drug sensitivity of recipient drug-sensitive cells by modulating drug-induced apoptosis [126]. Mechanistically, specific miRNA profiles, including those of miR-100 and miR-222, in drug-resistant breast cancer-derived exosomes have been investigated [126]. In addition, the transfer of miR-221/222 from tamoxifen-resistant breast cancer to tamoxifen-sensitive breast cancer by exosomes led modified p27 and estrogen receptor alpha expression and resulted in a drug-resistant response in the recipient cells [127].

Moreover, the crosstalk between cancer and the tumor microenvironment by exosomal miRNAs also modulates the growth and response to drugs of cancers [128]. In a recent study, increased exosomal miRNAs, such as miR-503, in neoadjuvant chemotherapy-treated endothelial cells exhibited an anti-breast cancer response [129]. Increased plasma miR-503 has been detected in breast cancer patients with neoadjuvant chemotherapy [129], suggesting that stromal cells modulate cancer development by releasing EV miRNAs in response to anticancer therapies.

### EVs in immunotherapy

Tumor-derived EVs in addition to tumor cells indeed participate in immunosuppression or immunostimulation in accordance with the development and progression of cancer [130–132]. Numerous studies have indicated that tumor-derived EVs mediate cancer development by inhibiting immune responses. For example, tumor-derived exosomes facilitated cancer immune evasion by triggering the downregulation the expression of natural killer group 2D, an activating receptor for natural killer (NK) cells, leading to NK effector functional defects [133]. Moreover, tumor-derived exosomes contained membrane-associated Hsp72, which interacts with myeloid-derived suppressor cells (MDSCs), reinforcing the signal transducer and activator of transcription 3-dependent immunosuppressive function of MDSCs [134]. Additionally, exosomes derived from Epstein-Barr virus-associated nasopharyngeal carcinoma exerted galectin-9, a ligand of T cell immunoglobulin and mucin domain-3 (TIM-3), to induce the apoptosis of mature T-helper type 1 lymphocytes [135]. In contrast, exosome-mediated apoptosis was blocked by both anti-Tim-3 and anti-galectin-9 antibodies [135]. Moreover, pancreatic cancer-derived exosomes also downregulated Toll-like receptor 4 and its downstream cytokines tumor necrosis factor-α and interleukin-12 in dendritic cells (DCs) via exosomal miR-203 [136]. Together, these comprehensive studies suggest that the ablation or inhibition of EV-mediated immune responses enhances the efficacy of immunotherapeutic anticancer therapies

Several studies indicated that exosomes secreted from antigen-presenting cells, such as B-cells, enable the induction of the immune response [137, 138]. Intriguingly, tumor-derived exosomes have been suggested to transfer tumor antigens to DCs, leading to potent CD8^+^ T-cell-dependent antitumor effects *in vivo* [139]. Clinical studies also suggested that tumor-derived exosomes isolated from ascites function as natural tumor rejection antigens [140]. Moreover, exosomes derived from NK cells expressed killing proteins, such as the Fas ligand and perforin molecules, suggesting anticancer activity [141]. An *ex vivo* study also indicated that healthy donor plasma-derived exosomes displayed NK markers with exosome-induced cytotoxicity [141]. Additionally, mast cell-derived exosomes were capable of facilitating the maturation of DCs and inducing immune responses [142]. Indeed, the concept of the exosome-elicited immune response has been under evaluation in a phase I clinical trial for the immunization of patients against melanoma by using autologous exosomes with MAGE 3 (melanoma-associated antigen 3) peptides [143]. In a phase I clinical trial, DC-derived exosomes loaded with cancer antigen induced anticancer immune responses [144]. Furthermore, exosomes from interferon-γ-maturated DCs were generated to facilitate anticancer immune responses in a phase II clinical trial [145]. Together, these studies highlight the feasibility of EV-based anticancer immunotherapy.

### EV-based targeted delivery

Given their cell- and tissue-tropic features, EVs are thought to be ideal therapeutic carriers for anticancer targeted therapy. Typically, in addition to carrying bioactive luminal cargos, EVs contain cell-cell and cell-ECM adhesion receptors on their surfaces to recognize distinct receptors of their target cells and tissues. For example, tetraspanin-8-expressed exosomes preferentially target CD11b/CD54-positive cells [146]. Interestingly, exosomes expressing modified receptors, such as tetraspanins, fused with specific candidate proteins displayed enhanced cell targeting [147]. Likewise, the expression of a fusion protein containing the αv integrin arginyl-glycyl-aspartic acid (RGD) peptide and an exosomal transmembrane protein, such as lysosome-associated membrane glycoprotein 2b (Lamp2b), allowed dendritic cell-derived exosomes to target αv integrin-positive cancers [148]. Small interfering RNA (siRNA)-carrying exosomes containing a fusion protein between central nervous system–specific rabies viral glycoprotein peptide and Lamp2b specifically targeted the acetylcholine receptor of neurons, resulting in brain-specific gene knockdown *in vivo* [149]. Since cyclic (Arg-Gly-Asp-D-Tyr-Lys, RGDyK) peptide, c(RGDyK), has a high binding affinity with integrin αvβ3 on cerebral vascular endothelial cells, c(RGDyK)-conjugated exosomes loaded with curcumin were specifically directed toward the lesion-containing region of the ischemic brain, where they then ameliorated inflammatory responses and apoptosis [150].

In light of the EV-mediated transfer of biological molecules, magnet-conjugated transferrin bound to transferrin receptor-expressed blood exosomes has been shown to preferentially target magnets surrounding cancer cells, followed by the inhibition of cancer development [151]. Furthermore, engineered glycosylation prevented the proteolytic degradation of exosomal-targeting ligands, suggesting the high stability and efficient targeting of glycosylated exosomes [152]. Due to the CD47-mediated protection of exosomes from phagocytosis, cell-derived exosomes carrying siRNAs exhibited enhanced efficacy in targeting oncogenic KRAS in a CD47-dependent manner [153]. Taken together, these studies suggest that EV-based targeted delivery, particularly with some modifications, is a highly efficacious alternative to cancer therapies.

### EVs as a therapeutic drug delivery system

Drug delivery is a critical determinant for the efficacy of clinical therapeutic treatment. As discussed above, EVs have emerged as a novel and promising drug delivery technology with the advantage of precise targeting, prolonged stability and controllable release. Despite the popularity of synthetic liposomes and polymeric nanoparticles in drug delivery [112, 154, 155], the instability and low biocompatibility of synthetic liposomes and polymeric nanoparticles, respectively, give rise to a degree of toxicity and lower efficacy in terms of clinical utility [156, 157]. In contrast, EVs exhibit biocompatibility, low toxicity, high drug delivery efficacy, specificity, and stability [158, 159]. More specifically, the lipid bilayer harbors many unique integral proteins with various posttranslational modifications that allow EVs to serve as a protective shelter for the sustainable release of anticancer drugs or cancer suppressors and to evade degradation and immune responses [160]. As a result, in human body fluids, EVs are proper nucleic acid drug (e.g., siRNAs or miRNAs) carriers. Consistently, anti-miR-9 delivered by mesenchymal stem cell-derived exosomes to glioblastoma multiforme cells reversed the expression of the multidrug transporter and sensitized the glioblastoma multiforme cells to chemotherapy drugs [161]. Additionally, exosomes can efficiently deliver microRNAs, such as let-7a, to breast cancer cells overexpressing EGFR, inhibiting cancer development *in vivo* [162]. Alternatively, the use of exosomes to deliver small molecules to treat cancers and other diseases has been demonstrated as well. For example, exosomes with encapsulated anti-inflammatory drugs, such as curcumin, exhibited increased solubility, stability, and drug bioavailability *in vitro* and *in vivo* [163].

#### EV-based therapy in brain disease

The blood-brain barrier (BBB) is a major obstacle for drug delivery to the central nervous system [164]. To mediate the delivery of misfolded proteins between neurons in neurodegenerative diseases [165], nanosized exosomes are presumably favorable for delivering agents/drugs across the BBB. Although nanoformulations are employed to improve the permeability of drugs across the BBB, toxicity and reticuloendothelial system-mediated or mononuclear phagocyte-mediated drug clearance both hinder the efficacy of man-made nanoformulations in treating diseases including cancers [166]. In contrast, EVs, which are naturally produced by cells, are guaranteed to exhibit biocompatibility and low antigenicity [158], which highlights the potential ability of EVs in treating brain disease, such as neurodegenerative diseases or brain cancers. Eventually, an exosome-based delivery system for antioxidants, such as catalase, that have a beneficial effect on patients with Parkinson’s disease will be approved [167]. Specific biological molecules must be used to cross the BBB or target brain tissue, and exosomes derived from brain endothelial cell displayed specific homing proteins that gave rise to increased transport across the BBB [168]. Thus, employing brain endothelial cells-derived exosomes to carry anticancer drugs, such as doxorubicin, is applicable for the suppression of brain cancer development [168].

#### Improvements in EV-based therapy

Nevertheless, some technical limitations in terms of the use of EVs as a drug delivery system, in particular, the efficiency of loading agents/drugs into EVs, are issues that remain to be resolved. Presumably, highly membrane-permeable small agents/drugs can easily be loaded into exosomes following incubation [169]. However, the loading of membrane-impermeable drugs, such as macromolecular drugs, siRNAs, and small DNAs, into exosomes using chemical approaches, such as temperature switching or detergents, is problematic. Recently, exosomes loaded with membrane-impermeable candidate nucleic acids or protein agents/drugs were produced by the pre-overexpression of these candidates in donor cells [161]. Alternatively, physical transfection methods, such as electroporation or liposome-mediated transfection, have been utilized to package membrane-impermeable agents/drugs into exosomes [170]. Unfortunately, the low efficiency of loading agents/drugs into exosomes through the use of membrane-permeable reagents (i.e., liposomes) remains unimproved [171]. Alternatively, several studies indicated that artificial exosome mimetics could be substitutes for exosome-based drug delivery [159]. Reportedly, exosome-mimetic nanovesicles loaded with chemotherapeutic drugs, such as doxorubicin, could target malignant cancers *in vivo* [172].

## Conclusions

Cancer development is an evolving, dynamic and highly regulated process associated with the tumor microenvironment and even distant tissues. Given the participation of EVs in local and systemic intercellular communication, the essential roles of EVs in the regulation of cancer progression and malignancy have been highlighted in the past decade, although EVs were observed more than three decades ago. Until now, the biogenesis and heterogeneity of EVs, the regulatory mechanisms of diverse cargo packaging into EVs, and the *in vivo* functionality of tumor-derived EVs have remained largely unknown. Nonetheless, numerous studies have provided valuable information, such as detailed EV cargo profiles; unique EV biomarkers/signatures for the early detection, diagnosis and treatment of cancers; and powerful methods to isolate EVs from cell culture conditioned media or body fluids. These efforts provide the impetus for the promising applications of EVs in disease management and emphasize the importance of EV biology in precision medicine.

The molecular features of cancers change dynamically during cancer development; following anticancer therapy, tumor-derived EVs reflect the real-time state of cancer cells and allow the monitoring of disease progression. As a result, in addition to their diagnostic, predictive, and prognostic utilities, tumor-derived EVs serve as novel anticancer targets. However, optimal and/or standardized methods for EV isolation, storage, and characterization are required for basic research and clinical standardization in the diagnosis and treatment of cancer and other diseases. Notably, a guideline for EV studies suggested by the ISEV entitled“Minimal Information for Studies of Extracellular Vesicles 2018 (MISEV2018)” [104] was discussed and published to provide comprehensive mandatory, mandatory if applicable and encouraged considerations. Nevertheless, any new guidelines will be updated in accordance with emerging studies and discoveries in EVs.

Multiple aspects of EVs in cancer biology have been indicated, which sheds a novel light in understanding the tumor microenvironment involved in cancer development and the potential uses of EVs in cancer management. As a result, EVs are an authentic key mediator in cancer biology.

## Abbreviations

AF4: Asymmetrical-flow field-flow fractionation
Alix: Alpha-1,3/1,6-mannosyltransferase (ALG2)-interacting protein X
BBB: Blood-brain barrier
CD: Cluster of differentiation
cfDNAs: Cell free DNAs
CTCs: Circulating tumor cells
CXCR4: C-X-C chemokine receptor type 4
DCs: Dendritic cells
DNA: Deoxyribonucleic acid
dsDNAs: Double-stranded DNAs
ECMs: Extracellular matrixes
EGFR: Epidermal growth factor receptor
EM: Electron microscopy
EMT: Epithelial-mesenchymal transition
EV: Extracellular vesicle
exoDNAs: Exosomal DNAs
HSP: Heat shock protein
ILVs: Intraluminal vesicles
ISEV: International society for extracellular vesicles
Lamp2b: Lysosome-associated membrane glycoprotein 2b
let-7: Lethal-7
MAGE 3: Melanoma-associated antigen 3
MALS: Multi-angle light scattering
MDSCs: Myeloid-derived suppressor cells
miRNAs: microRNAs
MISEV2018: Minimal information for studies of extracellular vesicles 2018
MMP: Matrix metallopeptidase
mRNAs: Messenger ribonucleic acids
MVBs: Multivesicular bodies
NF-κB: Nuclear factor kappa-light-chain-enhancer of activated B cells
NK: Natural killer
NTA: Nanoparticle tracking analysis
oncomiRs: Oncogenic microRNAs
RANK: Receptor activator of nuclear factor-κB
RANKL: Receptor activator of nuclear factor κB-ligand
RGD: Arginyl-glycyl-aspartic acid
RGDyK: Arg-Gly-Asp-D-Tyr-Lys
siRNA: Small interfering RNA
TAMs: Tumor-associated macrophages
TCA: Tricarboxylic acid
TGF-β: Transforming growth factor β
TIM-3: T cell immunoglobulin and mucin domain-3
TMPRSS2:ERG: transmembrane protease serine 2:transforming protein ERG
TSG101: tumor susceptibility gene 101
UTRs: untranslated regions
UV: ultraviolet
Wnt: wingless/integrated
